# Effects of collaboration on the foreign language side effect: evidence from Chinese international students using a dual-task paradigm

**DOI:** 10.1007/s10339-026-01340-w

**Published:** 2026-03-24

**Authors:** Makito Hirami, Qichao Song, Yufei Wang, Daisuke Fujiki

**Affiliations:** 1 Graduate School of Humanities and Social Sciences, Hiroshima University, Hiroshima, Japan; 2 School of Japanese and International Studies, Beijing Centre for Japanese Studies, Beijing Foreign Studies University, Beijing, China

**Keywords:** Foreign language side effect, Collaboration, Thinking ability, Dual-task paradigm

## Abstract

When using a second language in problem-solving, “linguistic processing” and “thinking” occur simultaneously, consuming cognitive resources and reducing thinking ability compared to using a native language (i.e., foreign language side effect; FoLSE). In collaborative settings, cognitive load may increase due to the need to listen to others. This study examines the impact of collaboration on FoLSE. We employed a dual-task paradigm with a thinking task (calculation problems) to assess thinking ability and a language task (word chain games) to stimulate linguistic processing. Chinese international students (*N* = 82) participated in either a collaboration group (pairs or trios, alternating language tasks) or an individual group. The results, contrary to expectations, suggested that collaboration may reduce FoLSE. Specifically, when collaborating in a trio, the interference rate when using a foreign language was similar to using a native language. Thus, collaboration appears to mitigate the foreign language effect. The findings may inform the design of collaborative learning environments and multilingual workplace communication strategies aimed at reducing the FoLSE and enhancing cognitive efficiency.

## Introduction

In the educational field, there is an increasing emphasis on collaborative problem-solving, with international students often expected to solve problems through communication in a second language (L2). For example, there are reports that insufficient language proficiency can hinder classroom communication and problem-solving in group tasks (Zhang and Mi 2010). Previous research has shown that collaborative interaction among L2 learners can support language development and facilitate problem-solving by allowing learners to pool cognitive resources and negotiate meaning (Swain and Lapkin 1998). Moreover, pair and group work in L2 writing tasks have been found to generate more language-related episodes and lead to more accurate and complex language use compared to individual work (Storch 2011).

Takano and Noda (1993) and Takano and Yagyu (2021) explained that problem-solving requiring communication involves the simultaneous processing of both “linguistic processing” and “thinking.” “Linguistic processing” refers to the handling of verbal input and output, including phonetic analysis in comprehension and linguistic planning, sentence generation, articulation, and self-monitoring in production. In contrast, “thinking” involves semantic processing not directly tied to verbal forms, such as making inferences, evaluating coherence and validity in listening, or constructing meaning, retrieving knowledge, and interpreting non-verbal cues in speaking (Takano and Yagyu 2021).

When these two cognitive tasks are carried out in parallel, there is a possibility that linguistic processing and thinking will interfere with each other. It is widely recognized that the simultaneous execution of two cognitive tasks often results in interference, leading to a decline in performance (e.g., Leone et al. 2017; Norman and Bobrow 1975). In particular, when using a foreign language in such situations, the cognitive resources dedicated to “linguistic processing” can detract from those available for thinking, leading to a decline in cognitive performance compared to using one’s native language (Takano and Yagyu 2021). This phenomenon is referred to as the “foreign language side effect (FoLSE).” Interference occurs even when using one’s native language; in one’s native language, linguistic processing is highly automated (e.g., Liu and Cao 2016), so the interference with parallel thinking is small. However, in the case of a foreign language that is not as well-mastered as one’s native language, linguistic processing is not highly automated, and the interference with thinking is thought to be greater than in the case of one’s native language.

## Literature review

### Effects associated with the cognitive costs of foreign language use

Foreign language use entails certain cognitive costs, which can, in turn, influence a range of behavioral and cognitive outcomes. In recent years, an emerging body of research has highlighted the foreign language effect (FLE), which posits that using a foreign language can, in certain contexts, change decision-making by reducing emotional biases and promoting more deliberate, analytical thinking (e.g., Costa et al. 2017; Jiao et al. 2025; Purpuri et al. 2025). For example, Keysar et al. (2012) demonstrated that L2 users are less susceptible to classic decision-making biases, such as the framing effect. Similarly, Costa et al. (2017) argued that using a foreign language fosters reflective reasoning, partly due to the psychological distance it creates from emotionally salient content. Supporting this notion, Ivaz et al. (2016) found that emotional processing related to the self is attenuated in L2, suggesting a reduction in emotional resonance.

Moreover, Hadjichristidis et al. (2017) proposed that while foreign language use may initially impose a higher cognitive load, it may simultaneously facilitate more rational responses by reducing intuitive biases that interfere with analytical reasoning. Extending this line of inquiry, Hadjichristidis et al. (2019) reported that L2 use alters judgment and decision-making patterns by attenuating culturally ingrained and emotionally driven biases.

Additionally, Hayakawa et al. (2017) demonstrated that individuals using a foreign language make more utilitarian moral judgments, suggesting that the cognitive demands and emotional distance inherent in L2 processing shift decision-making toward more deliberative reasoning. Building on this, Privitera et al. (2023) found that the magnitude of the moral foreign language effect depends on prior language experience: individuals with greater exposure to foreign language use showed a smaller shift toward utilitarian judgments, implying that familiarity with foreign language processing can mitigate its cognitive cost. Furthermore, Privitera (2024) showed that individual differences in executive control predict the extent of the moral foreign language effect, with stronger cognitive control associated with smaller native language (L1)–L2 differences in moral decision-making. Taken together, these studies suggest that the increased cognitive demands of foreign language use modulate reasoning and moral judgment by engaging executive control resources and reducing emotional interference.

### Cognitive side effects of foreign language use

The aforementioned studies suggest that the cognitive load associated with foreign language use can alter patterns of decision-making and moral judgment. At the same time, several studies have indicated that the increased cognitive demands of using a foreign language may instead impair overall performance (e.g., Takano and Noda 1993; Wen et al. 2025). To disentangle the effects of language use from general declines in thinking ability, researchers have employed dual-task paradigms to demonstrate the FoLSE (e.g., Lee et al. 2016; Takano et al. 2003). Takano and Noda (1993) introduced the concept of FoLSE, which refers to a temporary reduction in thinking ability during the processing of a foreign language. Importantly, this reduction is not due to linguistic difficulty per se but rather to the cognitive interference induced by the additional processing load required for foreign language use. In their dual-task paradigm, participants completed a nonlinguistic cognitive task (e.g., mental calculation or spatial reasoning) while simultaneously performing a linguistic task (e.g., sentence verification) in either their L1 or a foreign language. Performance on the cognitive task significantly deteriorated when the linguistic task was conducted in L2, even though the cognitive task itself did not involve language. This provided robust evidence for nonlinguistic cognitive interference specifically attributable to foreign language processing.

Takano and Yagyu (2021) extended the paradigm by investigating whether FoLSE also manifests in contexts that involve inner speech—that is, silent verbal reasoning conducted in the mind. This line of inquiry is crucial for understanding whether FoLSE reflects a general impairment in cognitive control or whether it also disrupts language-based cognitive functions. In their study, Japanese university students with limited English proficiency performed reasoning tasks in their native language (Japanese) while simultaneously engaging in a sentence verification task in either Japanese (L1 condition) or English (L2 condition). These tasks included categorical syllogisms, sentence completion, and analogical reasoning, all of which require active inner speech for successful performance. The results showed a significant decline in reasoning performance under the L2 condition, confirming that FoLSE persists even when the primary task is conducted in the native language and relies heavily on inner speech. These findings suggest that processing a foreign language imposes a cognitive load that interferes with concurrent verbal thinking, even when such thinking is carried out in the native language. This supports the hypothesis that FoLSE arises not merely from linguistic unfamiliarity but from a deeper disruption of cognitive resources involved in inner speech and executive functioning.

From a practical standpoint, the study has implications for bilingual individuals working or studying in environments that require frequent mental switching between languages. It highlights the cognitive cost associated with managing dual language systems and underscores the importance of designing bilingual tasks with sensitivity to potential FoLSE effects.

### Mechanisms underlying the FoLSE hypothesis

The mechanisms underlying the FoLSE can be understood in terms of cognitive load theory (Kirschner 2002; Paas et al. 2003), working memory (WM) models (Baddeley 2012), and cross-modal attention frameworks (Kunar et al. 2008). For example, the cross-modal attention model helps explain performance declines observed in dual-task paradigms involving visuospatial and linguistic tasks (e.g., Almor 2008), even when the two tasks appear to engage separate cognitive systems. Kunar et al. (2008) compared participants’ performance on a multiple object tracking (MOT) task across three conditions: novel word generation, shadowing, and a no-secondary task control. While shadowing did not impair MOT performance, novel word generation significantly reduced it. These findings suggest that in communicative contexts, individuals must manage limited cognitive resources to simultaneously engage in both linguistic processing and thinking. As linguistic processing cannot be fully separated from general cognitive processing, such interference may have minimal impact on performance when cognitive resources are sufficient or may be effectively managed. However, when cognitive resources are constrained, interference intensifies, leading to reduced performance. As foreign language processing imposes greater cognitive demands than native language processing, this interference is typically stronger in a foreign language context, giving rise to the FoLSE.

Although the term “FoLSE” itself is still emerging, several studies have demonstrated that L2 processing can tax cognitive resources, particularly in real-time language comprehension. Tyler (2001), for example, examined how topic knowledge modulates WM demands in native and nonnative listeners during auditory comprehension. Participants listened to the ambiguous “Washing Clothes” text (Bransford and Johnson 1972) while performing a concurrent arithmetic task. WM load was indexed by declines in arithmetic accuracy relative to single-task baselines. Results showed that native listeners maintained stable performance regardless of whether they had access to the topic, while nonnative listeners exhibited significantly greater WM interference when topic cues were absent. These findings suggest that L2 listeners—due to limited bottom-up decoding efficiency—must rely more heavily on top-down schematic knowledge to construct situation models, thereby increasing cognitive load under ambiguity.

This aligns with broader psycholinguistic findings that foreign language use amplifies task difficulty in complex reasoning or memory-intensive conditions. Hadjichristidis et al. (2017) argued that while foreign language processing may suppress intuitive or emotional reactivity, it also slows lexical retrieval and semantic integration, especially in time-sensitive or cognitively taxing tasks. Similarly, Ivaz et al. (2016) found that emotional resonance is attenuated in L2, possibly limiting the facilitative role emotion plays in memory encoding and retrieval.

### Factors that modulate FoLSE

The magnitude of the FoLSE is not uniform across contexts or individuals; rather, it is modulated by several key factors, notably the degree of similarity between the native and foreign languages, as well as the speaker’s proficiency in the foreign language.

From the perspective of cross-linguistic similarity, Takano and Noda (1995) proposed the linguistic similarity hypothesis, suggesting that the more structurally similar a foreign language is to a speaker’s L1, the smaller the cognitive interference. Their dual-task experiments demonstrated that grammatical similarity between L1 and L2 significantly attenuates the FoLSE: German and Japanese speakers using English, and Korean and English speakers using Japanese, all showed reduced interference when the target language was grammatically closer to their native language.

Expanding on this, Wen et al. (2025) further explored the relative impact of syntactic versus lexical similarity by comparing the FoLSE in native Chinese speakers using English or Japanese. While both English and Chinese follow an SVO word order, they differ significantly in their writing systems—English utilizes an alphabetic system, whereas Chinese utilizes a logographic system. This leads to considerable differences in lexical usage. In contrast, although Chinese and Japanese differ syntactically (SOV vs. SVO), they exhibit considerable orthographic and lexical overlap, primarily through the shared use of Chinese characters (Kanji), with many cognate words across the two languages. This suggests that, in terms of syntactic processing, Chinese and English are more closely aligned, while Chinese and Japanese exhibit greater similarity in lexical access. Wen et al. hypothesized that, if Chinese speakers experience more pronounced FoLSE when using Japanese, the syntactic processing costs are higher. Alternatively, if the FoLSE is more prominent when using English, lexical access plays a more significant role. The findings indicated a stronger FoLSE when participants used English as compared to Japanese, suggesting that lexical dissimilarity imposes a greater cognitive burden than syntactic similarity. This study also provided valuable insight into the possibility that lexical-level language processing may trigger more pronounced FoLSE, thereby contributing to a deeper understanding of its underlying mechanisms.

In addition to cross-language structural similarity, individual differences in language proficiency also play a decisive role in modulating FoLSE. Yang and Inoue (2017) examined how Japanese proficiency influenced FoLSE among Chinese international students, all of whom had passed the JLPT N1. Using a dual-task paradigm involving listening comprehension and mental calculation, they found that higher Japanese proficiency was significantly associated with reduced interference, whereas length of stay in Japan and academic background (e.g., Japanese-major status) had no significant effect. These results underscore that higher foreign language proficiency can reduce the executive demands associated with language processing, thereby mitigating the extent of FoLSE.

### Objectives the study

As reviewed above, the magnitude of the FoLSE is influenced by both individual differences in language proficiency and the inherent characteristics of the foreign language being used (e.g., Wen et al. 2025; Yang and Inoue 2017). However, an important contextual factor has been overlooked: the social setting in which the foreign language is employed. Notably, all prior studies of the FoLSE have focused exclusively on individual task settings (e.g., Takano and Yagyu 2021), where participants independently engaged in reasoning or problem-solving tasks while using a foreign language. In contrast, real-world cognitive activities—particularly in academic or workplace environments—often involve collaborative problem-solving and communication, frequently in a non-native language. In such contexts, individuals must not only manage their own cognitive load but also interact with others in a foreign language, potentially intensifying resource competition and cognitive interference.

To address this gap, the present study investigates the effects of collaboration on the magnitude of the FoLSE, specifically in situations where there is a high demand for foreign language use. Previous research has not explored how collaboration affects the FoLSE, and by examining how the presence of others impacts this phenomenon, we aim to gather more ecologically valid data from real problem-solving contexts.

## Experiment 1

### Hypotheses of experiment 1

To address the objectives of the study, we first compared the FoLSE in collaborative problem-solving situations (i.e., pairs of international students working together) versus individual problem-solving situations. Specifically, we examined interference rates under both native and foreign language conditions using a dual-task paradigm. This paradigm simultaneously engages participants in a language processing task and a cognitive reasoning task (i.e., mental arithmetic), allowing us to assess potential declines in thinking performance while processing language.

Consistent with previous research (e.g., Takano and Noda 1993; Takano and Yagyu 2021), we define linguistic processing as operations directly related to language input and output (e.g., reading aloud or verbal repetition), and thinking as cognitive operations not directly tied to language input/output (e.g., mental calculation). Importantly, we do not conceptualize these as entirely separate processes; rather, we consider them partially overlapping yet distinguishable components of cognitive activity. In natural language use, linguistic processing and thinking typically co-occur, and our experimental design was intended to simulate this interaction under controlled conditions.

In collaborative situations, participants are required not only to verbalize their own thoughts but also to listen to and understand the contributions of others (Boiteau et al. 2014). This increases the cognitive resources required for linguistic processing, which is expected to intensify the FoLSE. Prior studies have demonstrated that engaging in either listening or speaking can lead to decrements in performance on concurrent visuomotor tasks, suggesting that both modalities place significant demands on cognitive resources (Kubose et al. 2006). Consequently, we predict that collaborative efforts will result in a stronger FoLSE compared to individual efforts, leading to a greater decline in thinking ability.

Specifically, since the cognitive load when using a foreign language is greater than when using a native language (Nawal 2018), we expect that the impact of collaboration will be stronger. Thus, we hypothesize that there will be no difference in the decline of thinking ability when using one’s native language between collaborative and individual efforts, but the decline in thinking ability when using a foreign language will be greater in collaborative efforts than in individual efforts.

### Methods

#### Participants

The sample consisted of 40 Chinese international students (29 females and 11 males; mean age = 25.3 years) enrolled at a Japanese national university. Participants were recruited via an online community for international students at the university with which the first author is affiliated, using flyers outlining the purpose and procedure of the experiment. Upon completion of the experiment, participants received a ¥1,000 QUO card as compensation. All participants had attained the highest level of the Japanese Language Proficiency Test (JLPT N1), which corresponds approximately to the C1 level of the Common European Framework of Reference for Languages (CEFR). Participants were first paired and then randomly assigned either to a collaboration condition, in which 10 pairs (20 participants) completed the tasks collaboratively, or to an individual condition, in which 20 participants completed the tasks individually.

To ensure comparability between the two groups (i.e., individual vs. pair) in terms of Japanese proficiency (self-rated listening, speaking, reading, and writing skills), years of Japanese study, and frequency of Japanese–Chinese language use, we adopted the methodology of Li et al. (2020) and employed the Language History Questionnaire (LHQ3). To minimize participants’ cognitive load, only items directly relevant to these indicators were extracted from the LHQ3 to construct the post-experiment questionnaire. Statistical analyses (see Table 1) indicated no significant differences between the two groups in self-rated Japanese proficiency, duration of Japanese language learning, frequency of Japanese use, self-rated Chinese proficiency, or frequency of Chinese use [*t*(38) = 0.00–0.80, *ps* > .050, *d* = 0.00–0.25]. No significant differences were found in age [*t*(38) = 0.25, *p* = .801, *d* = 0.08] or gender distribution [χ*²*(1) = 0.50, *p* = .479, *w* = 0.11] between the groups. Additionally, all participants reported significantly higher proficiency and usage frequency in Chinese than in Japanese [*t*(39) = 12.59–14.65, *ps* < .001, *d* = 1.99–2.32], indicating that they were unbalanced bilinguals (e.g., Song et al. 2023).

**Table 1:** Demographic and language background information of participants (Experiment 1)

	Group		*N*	*M*	*SD*	Statistical comparison
Years of Japanese study	Individual		20	6.70	2.44	*t*(38) = 0.12, *p* = .903, *d* = 0.04
	Pair		20	6.80	2.50	
Self-rated Chinese proficiency	Individual		20	6.10	0.98	*t*(38) = 0.05, *p* = .963, *d* = 0.01
	Pair		20	6.09	0.69	
Self-rated Japanese proficiency	Individual		20	4.64	0.95	*t*(38) = 0.00, *p* = 1.000, *d* = 0.00
	Pair		20	4.64	0.80	
Frequency of Chinese use	Individual		20	6.21	0.71	*t*(38) = 0.14, *p* = .889, *d* = 0.04
	Pair		20	6.24	0.79	
Frequency of Japanese use	Individual		20	3.11	0.96	*t*(38) = 0.80, *p* = .429, *d* = 0.25
	Pair		20	2.86	1.01	
Age (years)	Individual		20	25.20	2.76	*t*(38) = 0.25, *p* = .801, *d* = 0.08
	Pair		20	25.45	3.43	
Gender distribution	Female	Individual	13	—	—	χ*²*(1) = 0.50, *p* = .479, *w* = 0.11
		Pair	16	—	—	
	Male	Individual	7	—	—	
		Pair	4	—	—	

Individual group = participants performed the word-chain game individually. Pair group = two participants performed the word-chain game collaboratively. *M,* mean; *SD,* standard deviation.

#### Materials

1. Calculation Task

Calculation problems involving addition and subtraction of two-digit natural numbers (e.g., 38 + 61=?, 83 − 72=?) used in Yang and Inoue (2017) were used as a thinking task to measure thinking ability. This task does not involve linguistic processing; as such, it reflects thinking ability. To control for task-related effects in the within-participant design, three A4-sized sheets of paper were prepared with 72 calculation problems written on the front side.

2. Language Task

To encourage linguistic processing, we used word chain games in Japanese and Chinese. Word chain games are games in which players take turns saying different words beginning with the last letter of the word spoken by the other person. We used this task because it requires linguistic processing, can be conducted simultaneously with a calculation task, and can be carried out in a natural manner both collaboratively and individually. Moreover, because the word chain games prohibit the reuse of previously spoken words, participants in the collaborative condition are required to carefully attend to and comprehend their partners’ utterances. This feature allows the task to approximate a natural communicative context to some extent, making it well suited for examining the interactional aspects of collaboration.

Furthermore, Japanese and Chinese differ fundamentally in their writing systems: Japanese Hiragana is a phonetic script, whereas Chinese characters are logographic and typically carry both pronunciation and semantic information (Cai et al. 2025; Fei et al. 2022; Wen et al. 2025). In the Japanese task, decisions were based on the final Hiragana character, which usually represents only a syllable and has no tonal variation. In contrast, Chinese characters involve both tone and meaning, and the same syllable and tone can correspond to multiple characters, each with a distinct meaning. We were aware of this asymmetry and took measures to mitigate it. Specifically, in the Chinese version of the word chain games, participants were allowed to respond using homophones (same pronunciation) regardless of tone or character identity. That is, the follow-up word could start with any Chinese character that shares the same pronunciation (Pinyin) as the last character of the preceding word, even if the character or tone was different. This adjustment effectively shifted the focus of the task from semantic to phonological processing, making it more comparable to the Japanese task, in which decisions are purely based on sound.

Additionally, as the study employed a within-subjects design, all participants completed both the Chinese and Japanese tasks. This design helped control for individual differences and minimized the impact of structural asymmetries between the two languages on the overall results.

3. Vocabulary Test

The vocabulary test used to measure Japanese vocabulary skills was the Japanese Common Academic Word Test (Written) Ver. 2.51 (Tajima et al. 2018). This test consists of 75 vocabulary semantic choice questions (1 point each, for a total of 75 points). In each question, participants first see a Japanese word, followed by a sentence that uses the target word. Participants are required to choose the most appropriate option that aligns with the meaning of the target word in the given context. They must select their answer within 30 s, although they may skip any question if they are unsure about the word’s meaning.

#### Procedure

**Fig. 1:**
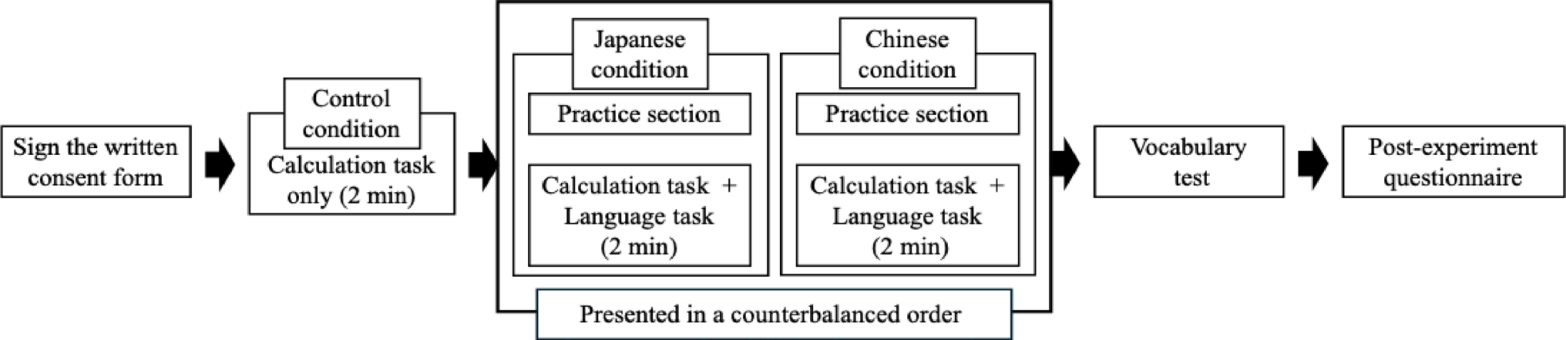
Experimental procedure. *Both the collaborative and individual groups followed the same procedure. In the word chain games, participants in the collaborative group took turns producing words in pairs, while those in the individual group generated words sequentially on their own. The Japanese and Chinese conditions were treated as within-subject variables, and their order was counterbalanced.

The experimental procedure is shown in Fig. 1. The experiment was conducted one participant (or pair) at a time in a single room at the university.

First, all participants provided written consent before the start of the study procedure.

Next, participants attempted three tasks, each lasting two minutes: a control condition, in which they worked only on the calculation task; a Japanese condition, in which they simultaneously performed the language task in Japanese and the calculation task; and a Chinese condition, in which they carried out the language task in Chinese while also working on the calculation task. Participants were instructed to solve calculation problems as quickly and accurately as possible in the calculation task. The control condition was completed first, and the order of the Japanese and Chinese conditions was counterbalanced. Three equivalent sets of calculation tasks were prepared (used in the control, Chinese, and Japanese conditions), each matched for difficulty. These sets were also controlled across groups to eliminate confounding effects.

**Fig. 2:**
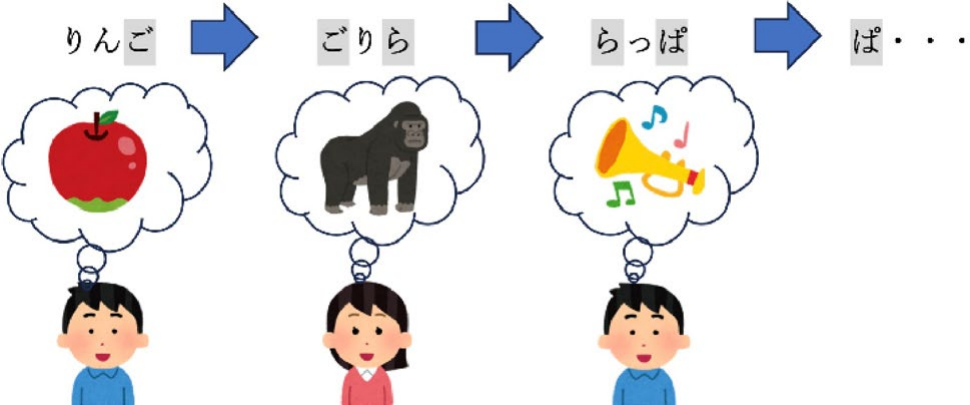
Examples of Japanese word chain games

**Fig. 3:**
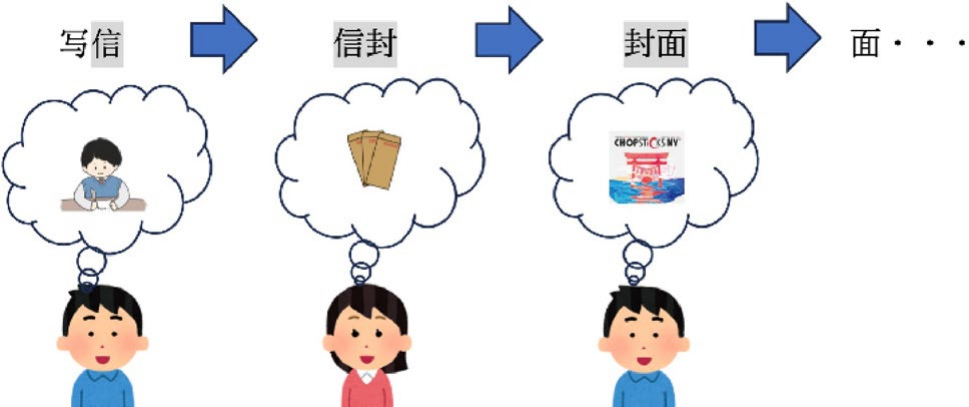
Examples of Chinese word chain games

Before starting the main tasks, participants practiced the word chain game in both the Japanese and Chinese conditions to confirm the rules. We explained the rules of the game (e.g., the same word cannot be used twice) with examples (Figs. 2 and 3). While the Chinese condition permits homophones with different characters and tones, a phonological constraint in Japanese word chain games forbids the use of words ending with “ん, n”, as no native Japanese words begin with this sound. We instructed that if it became too difficult to continue, they could restart by saying a new word.

Furthermore, to ensure that the words were spoken at a uniform pace, a timer sounded once every 5 s. Participants’ voices during the word chain games were recorded using a voice recorder.

Specifically, in the pair condition, participants were seated next to each other and alternated turns in producing words during the word chain game. Participants in the individual condition performed the word chain games alone (i.e., generating word chains by themselves), without collaboration. Aside from the absence of interaction, all other experimental settings were kept consistent with the collaborative conditions. This non-collaborative language task was essential to examine the effect of language use itself, independent of collaborative dynamics. Its inclusion allows for a direct comparison with collaborative conditions and thus serves as a crucial control group.

Finally, participants individually completed a vocabulary test. Using their own smart phones, they scanned a QR code and answered questions online and submitted their responses immediately on-site. To ensure that participants reported their scores accurately, they were asked to show the final score screen displayed on their device. Then they wrote down their finally displayed scores on a test form. Additionally, at the conclusion of the experiment, participants were asked to complete a post-experiment questionnaire (i.e., LHQ 3). To ensure response validity, they were also asked to indicate their honesty by checking an item stating, “I answered all items truthfully.”

### Results and discussion

#### Data processing

1. Vocabulary Test Scores

The mean scores for the vocabulary test were 66.20 (*SD* = 3.82) for the collaboration group and 67.40 (*SD* = 3.69) for the individual group. An independent-sample Welch t-test was conducted to confirm whether there was a difference between the collaboration and individual groups, but no significant difference was found (*t*(38.0) = 1.01, *p* = .319, *d* = 0.32).

2) Calculation and Language Task Scores

We scored the calculation task based on the number of correct answers to calculation problems and scored the language task based on the number of words spoken per person in word chain games. However, because the purpose of the language task was to apply a load on linguistic processing, even if the rules of the word chain game were not followed correctly, if words were uttered in an attempt to continue the word chain, they were counted. Calculation tasks and language tasks for the collaboration and individual groups are presented in Table 2.

**Table 2:** Scoring of calculation task and language task for collaboration group and individual group

	Calculation task			Language task	
	Collaboration group	Individual group		Collaboration group	Individual group
Control condition	35.05 (9.89)	35.30 (8.78)		−	−
Japanese condition	13.70 (8.18)	9.30 (5.92)		12.15 (1.73)	20.80 (4.62)
Chinese condition	16.70 (6.91)	15.35 (7.83)		19.15 (2.70)	30.90 (8.10)

Results are shown as mean (standard deviation).

3) Tabulation of FoLSE

As an index to measure the magnitude of the FoLSE, we calculated the rate of interference (*I*) between linguistic processing and thinking based on Takano and Noda (1993) using the following formula.

1 $$ I = \left[ {\left( {S - D} \right){\text{ }}/S} \right] \times 100\left( \% \right) $$

*S* is the number of correct answers to the calculation task in the control condition, and *D* is the number of correct answers in the Japanese or Chinese condition. Simultaneous engagement in linguistic processing and thinking can lead to interference when cognitive resources are depleted. Accordingly, the extent of cognitive decline is quantified using the *rate of interference*, which reflects the percentage reduction in thinking task performance when linguistic processing is involved, relative to a baseline condition without such processing. A simple comparison between native and foreign language performance would not adequately reflect this decrement, as it would conflate baseline differences with interference effects. The magnitude of the FoLSE can be assessed by comparing the rate of interference observed when tasks are performed in a foreign language versus in one’s native language. The larger the value of this rate of interference means the larger the FoLSE.

When we calculated the rate of interference as an index of the magnitude of the FoLSE, in the Japanese condition it was 60.30 (*SD* = 19.86) in the collaboration group and 72.14 (*SD* = 22.58) in the individual group, and in the Chinese condition it was 50.98 (*SD* = 18.44) in the collaboration group and 54.77 (*SD* = 26.41) in the individual group.

4) Language-Related Control Variables

To control for individual differences in language proficiency that could potentially affect cognitive performance during tasks, both objective and subjective measures of Japanese language ability were collected. The objective measure consisted of a vocabulary proficiency test administered on the day of the experiment, providing a reliable and task-relevant indicator of participants’ current language competence. Subjective measures included self-assessments of four core language skills (listening, speaking, reading, and writing). Correlation analyses revealed a modest positive relationship between the self-rated language proficiency and vocabulary test scores (*r* = .38, *df* = 38, *p* = .017), providing preliminary support for the convergent validity of the subjective ratings.

As the vocabulary test was the only objective measure obtained concurrently with the experimental task and was most directly related to the lexical demands of our language task, it was selected as the primary covariate for language proficiency in the mixed-effects model. This decision was also informed by considerations of model parsimony and statistical stability, particularly considering the available sample size, in accordance with the approach adopted by Song et al. (2025).

#### Effects on magnitude of the foreign language effect

To examine whether the magnitude of the FoLSE differed between collaborative and individual conditions, we conducted a linear mixed-effects model analysis with the rate of interference as the dependent variable. Compared with traditional ANOVA, linear mixed-effects models offer more robust estimates, accommodate covariates such as participants’ Japanese vocabulary proficiency, and appropriately account for the repeated-measures structure of the data, thereby enhancing the accuracy and interpretability of the results. The analysis was implemented using the GAMLj module (Gallucci 2019) in Jamovi (version 2.6.2), which internally relies on the glmer function from the lme4 package in R (R Core Team, 2024). Categorical predictors were coded using simple (treatment) contrasts, with the individual task and the Chinese conditions serving as reference levels (coded as 0). Vocabulary proficiency was mean-centered prior to analysis.

Consistent with recommendations to specify the maximal random-effects structure supported by the data (Barr et al. 2013), initial models included random intercepts and random slopes for within-participant predictors. However, models with random slopes failed to converge and produced warnings indicating unidentifiable random-effects parameters, likely owing to the limited number of observations per participant. Accordingly, the final model included random intercepts for participants only. Model specification was primarily constrained by convergence diagnostics and parameter identifiability; information criteria (AIC and BIC values) for the final convergent model are reported to facilitate transparency. The final model included angular-transformed FoLSE scores as the dependent variable, with fixed effects of centered vocabulary proficiency, group (0 = individual, 1 = collaboration), condition (0 = Chinese, 1 = Japanese), and their interaction. Participants were included as a random intercept. Residual diagnostics indicated no significant deviation from normality (see Table 3).

**Table 3:** Effects of collaboration or individual efforts on the rate of interference

Fixed effects parameter estimates							
Names	Effect	Estimate	*SE*	*df*		*t*	*p*
(Intercept)		0.67	0.04	37		17.86	< .001
Condition	1 − 0	0.18	0.04	38		4.60	< .001
Group	1 − 0	−0.14	0.08	37		−1.80	.080
Japanese vocabulary proficiency		−0.02	0.01	37		−1.65	.107
condition × group	1 − 0 × 1 − 0	−0.11	0.08	38		−1.42	.163

Random components							
Groups	Name	*SD*	Variance		*ICC*		
Participant	(Intercept)	0.20	0.04		0.58		
Residual		0.17	0.03				

Participants = 40; total observations = 80. *SE,* standard error; *df,* degree of freedom; *SD,* standard deviation; *ICC,* intraclass correlation coefficient. R^2^ marginal = 0.18; R^2^ conditional = 0.66. Models were estimated using restricted maximum likelihood (REML) with the BOBYQA optimizer as implemented in lme4 (via Jamovi). The optimal model is [FoLSE_transformed ~ group + condition + Japanese vocabulary proficiency + group × condition + (1 | participant)]. Tests for the normality of residuals indicated no significant deviation from normality (Kolmogorov–Smirnov = 0.11, *p* = .266; Shapiro–Wilk = 0.98, *p* = .163). AIC = 6.97; BIC = 47.23.

As shown in Table 3, a significant main effect of condition was observed, indicating a higher level of interference in the Japanese condition than the Chinese condition (*p* < .001). This finding confirms the presence of the FoLSE effect in the current experiment. In contrast, the main effect of group showed a marginal trend toward significance (*p* = .080), suggesting that collaboration may have helped mitigate interference relative to individual performance.

#### Discussion

In this experiment, we examined whether collaborative efforts result in a stronger FoLSE compared to individual efforts. Building on previous research (e.g., Yang and Inoue 2017), we designed an experimental task to investigate this relationship. The results indicated that the effects varied depending on the condition, with a significantly higher rate of interference in the Japanese condition than the Chinese condition. This finding suggests that L2 use imposes a greater cognitive load, intensifying competition for cognitive resources and ultimately leading to a stronger FoLSE.

However, contrary to our initial hypothesis, collaborative efforts appeared to reduce the rate of interference compared to individual efforts. This finding contradicts the prediction that “collaborative efforts will result in a stronger FoLSE compared to individual efforts, leading to a greater decline in thinking ability,” instead suggesting that collaboration may help mitigate declines in thinking ability. Nonetheless, given that the statistical results did not reach the conventional significance threshold, this finding alone is insufficient to conclusively determine whether collaboration reduces interference.

Moreover, the interaction effects were not significant, failing to support the prediction that there would be no difference in the decline in thinking ability when using one’s native language between collaborative and individual efforts, but the decline in thinking ability when using a foreign language would be greater in collaborative efforts than in individual efforts.

## Experiment 2

### Hypotheses of Experiment 2

In Experiment 1, the results contradicted our predictions. To gain a clearer understanding of the effects of collaboration on the FoLSE, we conducted a supplementary experiment (Experiment 2). While Experiment 1 focused on paired collaboration, we hypothesized that increasing the number of participants would clarify differences from individual efforts. In Experiment 2, we used trios for collaborative situations and conducted a reanalysis by integrating the newly collected data with that from Experiment 1. The trio condition is assumed to capture the distinctive characteristics of collaboration more effectively than the pair condition. Fundamentally, the key difference between individual and collaborative efforts lies in the requirement to attend to others’ verbal contributions in the latter. Therefore, increasing the group size from two to three members provides greater opportunities to process partners’ utterances, both to follow the previous word and to ensure that no word is repeated by any member, thereby enhancing the collaborative nature of the task.

Based on the initial findings, we predicted that collaboration, especially in trios, will result in a smaller decline in thinking ability than individual efforts. Additionally, we predicted that there will be no difference in the decline in thinking ability when using a native language between trio and individual efforts, but the decline when using a foreign language will be smaller in trio efforts than in individual efforts.

### Methods

#### Participants

We recruited 14 trios (42 participants; 25 females, 17 males; mean age = 24.8 years) for Experiment 2 from the same Japanese national university as in Experiment 1. All participants had passed the JLPT at the N1 level and were classified as advanced learners, consistent with the participant criteria in Experiment 1. A paired-sample *t*-test comparing bilingual proficiency and language use frequency revealed that participants reported significantly higher proficiency and usage frequency in Chinese than in Japanese (*t*(41) = 11.11–11.78, *ps* < .001, *d* = 1.73–1.84), confirming that they were unbalanced Chinese–Japanese bilinguals.

To assess comparability across experiments, we conducted a one-way ANOVA on participants’ self-rated bilingual proficiency, frequency of Japanese–Chinese language use, and duration of Japanese language learning across three groups (individual, pair, and trio). The results (see Table 4) showed no significant differences across these measures (*F*(2, 79) = 0.18–2.62, *ps* > .050, η² = 0.01–0.06), suggesting that the three groups were comparable in linguistic background. Similarly, no significant differences were found across groups in gender distribution (χ*²*(2) = 2.54, *p* = .327, *w* = 0.18) or age (*F*(2, 79) = 0.35, *p* = .707, η² = 0.01).

**Table 4:** Demographic and language background information of participants (Experiment 2)

	Group		*N*	*M*	*SD*	Statistical comparison
Years of Japanese study	Individual		20	6.70	2.44	*F*(2, 79) = 0.83, *p* = .438, η² = 0.02
	Pair		20	6.80	2.50	
	Trio		42	6.07	2.37	
Self-rated Chinese proficiency	Individual		20	6.10	0.98	*F*(2, 79) = 0.18, *p* = .840, η² = 0.00
	Pair		20	6.09	0.69	
	Trio		42	6.20	0.73	
Self-rated Japanese proficiency	Individual		20	4.64	0.95	*F*(2, 79) = 0.43, *p* = .652, η² = 0.01
	Pair		20	4.64	0.80	
	Trio		42	4.45	0.98	
Frequency of Chinese use	Individual		20	6.21	0.71	*F*(2, 79) = 2.62, *p* = .079, η² = 0.06
	Pair		20	6.24	0.79	
	Trio		42	5.74	1.11	
Frequency of Japanese use	Individual		20	3.11	0.96	*F*(2, 79) = 0.27, *p* = .762, η² = 0.01
	Pair		20	2.86	1.01	
	Trio		42	3.02	1.19	
Age (years)	Individual		20	25.20	2.76	*F*(2, 79) = 0.35, *p* = .707, η² = 0.01
	Pair		20	25.45	3.43	
	Trio		42	24.83	2.54	
Gender distribution	Female	Individual	13	—	—	χ*²*(2) = 2.54, *p* = .327, *w* = 0.18
		Pair	16	—	—	
		Trio	25	—	—	
	Male	Individual	7	—	—	
		Pair	4	—	—	
		Trio	17	—	—	

Individual group = participants performed the word-chain game individually. Pair group = two participants performed the word-chain game collaboratively. Trio group = three participants performed the word-chain game collaboratively. *M,* mean; *SD,* standard deviation. The main effect of frequency of Chinese use showed a trend toward significance (*p* = .079). However, post hoc comparisons indicated no significant differences among the three groups (*ps* > .100). The demographic and language background information from Table 1 is also included here for comparison purposes.

Taken together, these results support the homogeneity of participants across the two experiments. Participants were assigned to groups of three and completed the task under the trio collaboration condition. Upon completing the experiment, each participant received a ¥1,000 Quo card as compensation.

#### Materials

The same materials were used as in Experiment 1.

#### Procedure

The procedure was nearly identical to that used in Experiment 1. After providing written informed consent, participants engaged in the experimental tasks following the same sequence as before: they first completed a control condition involving only the calculation task, followed by Japanese and Chinese conditions that combined the calculation task with a language task. Each condition lasted two minutes. However, in the present experiment, participants completed the language task in trios, taking turns in a fixed sequence during the word chain game. Three participants were seated in a row. The task began with the person seated at one end, and the word chain proceeded in a predetermined sequence, with each participant responding to the previous word in turn. Seven trios completed the Japanese condition first, while the other seven trios completed the Chinese condition first to counterbalance the order. As in Experiment 1, a vocabulary test was administered at the end of the session, following completion of the main tasks. The same ethical considerations as in the Experiment 1 were applied.

### Results and discussion

#### Data processing

During data screening and verification, we discovered that one of the participants had previously taken part in Experiment 1. As a result, the entire trio that included this individual was excluded from subsequent analyses. None of the remaining 39 participants had participated in Experiment 1, and their data were included in the following analyses.

The mean score on the vocabulary test for the trio collaboration group was 65.13 (*SD* = 7.85). To assess comparability in language proficiency, we conducted a one-way ANOVA comparing vocabulary scores across the three groups. No significant differences were found (*F*(2, 76) = 0.92, *p* = .404, η² = 0.02), suggesting that participants in the collaboration (i.e., pair and trio) and individual groups had similar levels of Japanese vocabulary proficiency.

Consistent with Experiment 1, the vocabulary proficiency test was retained as the covariate for language ability in the statistical model, as it was the only objective measure administered concurrently with the experimental tasks. Following the same scoring procedure as in Experiment 1, we calculated the scores for the thinking and language tasks in the trio collaboration group (Table 5). The rate of interference, used as an index of the magnitude of the FoLSE, was 51.84 (*SD* = 16.93) in the Japanese condition and 43.12 (*SD* = 22.38) in the Chinese condition.

**Table 5:** Trio collaboration group thinking and language task scores

	Calculation task	Language task
Control condition	38.26 (10.44)	−
Japanese condition	18.05 (7.46)	8.46 (1.35)
Chinese condition	21.28 (9.06)	14.41 (1.67)

Results are shown as mean (standard deviation).

#### Effect of trio collaboration on the magnitude of the FoLSE

To examine whether the magnitude of the FoLSE differed between individual and collaborative conditions (i.e., pair and trio collaboration), we conducted a linear mixed-effects model analysis with the rate of interference as the dependent variable. Data from Experiment 2 were combined with those from Experiment 1 for reanalysis to increase statistical power and enable a more comprehensive comparison. The dependent variable was the angular-transformed FoLSE score. Fixed effects were group (0 = individual, 1 = pair collaboration, 2 = trio collaboration) and condition (0 = Chinese, 1 = Japanese), which were coded using simple (treatment) contrasts, as well as centered vocabulary test scores and the group × condition interaction.

Following the same analytical procedure as in Experiment 1, initial models included random intercepts and random slopes for within-participant predictors. However, models including random slopes did not converge and yielded warnings indicating unidentifiable random-effects parameters. Consequently, the final model included random intercepts for participants only. Model specification was therefore guided primarily by convergence diagnostics and parameter identifiability; model fit indices for the final convergent model are reported for reference. Residual diagnostics indicated no significant deviation from normality (see Table 6).

**Table 6:** Effects of individual, pair, and trio efforts on the rate of interference

Fixed effects parameter estimates						
Names	Effect	Estimate	*SE*	*df*	*t*	*p*
(Intercept)		0.62	0.03	75	24.44	< .001
Condition	1 − 0	0.15	0.03	76	5.22	< .001
group1	1 − 0	−0.12	0.07	75	−1.82	.079
group2	2 − 0	−0.23	0.06	75	−3.83	< .001
Japanese vocabulary proficiency		−0.01	0.01	75	−1.42	.300
group1 × condition	1 − 0 × 1 − 0	−0.11	0.08	76	−1.41	.166
group2 × condition	2 − 0 × 1 − 0	−0.13	0.07	76	−2.06	.054

Random components						
Groups	Name	* SD*	Variance	*ICC*		
Participant	(Intercept)	0.18	0.03	0.51		
Residual		0.17	0.03			

Participants = 79; total observations = 158. *SE*, standard error; *df*, degree of freedom; *SD*, standard deviation; *ICC*, intraclass correlation coefficient. R^2^ marginal = 0.19; R^2^ conditional = 0.60. Models were estimated using restricted maximum likelihood (REML) with the BOBYQA optimizer as implemented in lme4 (via Jamovi). The optimal model is [FoLSE_transformed ~ group + condition + Japanese vocabulary proficiency + group × condition + (1 | participant)]. Tests for the normality of residuals indicated no significant deviation from normality (Kolmogorov–Smirnov = 0.05, *p* = .878; Shapiro–Wilk = 0.99, *p* = .503). AIC = −4.01; BIC = 58.31.

Based on the results of the analysis, a significant main effect of condition was observed, with a higher level of interference in the Japanese condition than the Chinese condition (*p* < .001). Consistent with the findings from Experiment 1, the collaboration group showed a marginal trend toward reduced interference relative to the individual group (*p* = .079). Moreover, within the collaboration conditions, the trio group also exhibited a marginally lower level of interference compared to the pair group (*p* = .069). Notably, the trio collaboration group exhibited significantly lower interference than the individual group (*p* < .001).

Furthermore, the interaction between group and condition showed a marginally significant trend in the trio collaboration group (*p* = .054). Post hoc comparisons using Bonferroni correction revealed that, in the Japanese condition, the trio collaboration group exhibited significantly lower interference than the individual group (*t*(116.5) = 4.26, *p* < .001). In the Chinese condition, although the interference rate was still lower in the trio group, the difference was not statistically significant (*t*(116.5) = 2.38, *p* = .285). Additionally, the individual group demonstrated significantly greater interference in the Japanese condition than the Chinese condition (*t*(76.0) = 4.19, *p* = .001), whereas no such difference was observed in the trio collaboration group (*t*(76.0) = 2.49, *p* = .226).

#### Discussion

As Experiment 1 did not fully verify the specific effects of collaboration on FoLSE, we conducted Experiment 2 and integrated the data for reanalysis. The results indicated that, overall, performing language tasks in L2 Japanese demanded greater cognitive resources, further confirming that FoLSE also emerged in this context.

Notably, the rate of interference in the trio collaboration group was significantly lower than in the individual group, and showed a decreasing trend compared to the pair collaboration group. This finding supports the prediction that *collaborative efforts*,* especially in trios*,* will result in less of a decline in thinking ability than individual efforts.* Compared with the pair condition, a trio configuration in the word chain games provides each individual with fewer opportunities to speak while simultaneously increasing the need to attend to others’ utterances due to the prohibition on repeating previously used words. Although the comparison between the pair and individual conditions showed only a marginal trend, a statistically significant difference was observed between the trio and individual conditions. These findings indicate that introducing the trio condition effectively highlighted the distinctive characteristics of collaborative performance.

Furthermore, the trio collaboration group demonstrated a lower rate of interference than the individual group in both the Japanese and Chinese conditions, with the difference reaching statistical significance in the Japanese condition. Importantly, no significant difference in interference rates between the Japanese and Chinese conditions was observed within the trio collaboration group. These findings suggest that trio-based collaboration more effectively prevents declines in cognitive performance compared to working individually, thereby reducing the likelihood of FoLSE. Consequently, the prediction was supported that there is no difference in the degree of decline in thinking ability when using one’s native language between trio efforts and individual efforts, but the degree of decline in thinking ability when using a foreign language in trio efforts is smaller than in individual efforts.

Kirschner et al. (2011) argued that collaborative problem-solving is effective when the task is complex and exceeds an individual’s WM capacity, as information processing can be distributed among group members’ working memories. Known as the collective working memory effect, this phenomenon is situated within the framework of distributed cognition (Hutchins 1995). It describes how collaborative information processing enables groups to handle tasks that surpass the WM capacity of any individual member. The effect tends to be more salient in complex tasks, offering a theoretical rationale for the effectiveness of collaborative learning. In this study, participants engaged in a dual-task paradigm that required complex cognitive processing (e.g., Esmaeili Bijarsari 2021). While the collaborative condition imposed a unique cognitive demand—requiring participants to listen to their partner’s utterances—it may have led to improved performance relative to the individual condition, as the responsibility for the language task could be shared. This division of labor may have alleviated individual cognitive load. Kirschner et al. (2011) also noted that collaboration is effective when its benefits outweigh the costs of maintaining the group. Although the dual-task situation imposed a high cognitive load, the collaborative condition may have helped distribute this load more effectively across participants. As the groups consisted of individuals who were familiar with one another, the costs of maintaining the group were likely low (e.g., Adams et al. 2005). Thus, the benefits of collaboration likely outweighed these costs, reducing the FoLSE.

In summary, our experimental hypothesis was largely supported, indicating that collaborative efforts can, to some extent, mitigate FoLSE. This suggests that collaboration may serve as a compensatory mechanism that alleviates the cognitive demands imposed by foreign language processing, thereby reducing the impact of FoLSE.

## General discussion

This study examined the effect of collaboration on the FoLSE. Previous studies have indicated that the use of a foreign language involves certain cognitive costs, which can influence a variety of outcomes (Costa et al. 2017; Ivaz et al. 2016; Jiao et al. 2025; Keysar et al. 2012; Purpuri et al. 2025). In the present study, we focused on the cognitive disadvantages associated with foreign language use, particularly those arising from increased cognitive load. The results showed that the FoLSE occurred even in collaborative problem-solving situations involving communication in a foreign language. Additionally, while the interference rate was higher in the Japanese condition than in the Chinese condition during individual efforts, no such difference was observed in the trio collaboration condition. This suggests that collaborating in a trio not only reduced the interference rate but also prevented an increase in interference, especially when using a foreign language. Contrary to initial expectations, collaborative efforts appeared to reduce the FoLSE compared to individual efforts.

The language task in this study, word chain games, was used to impose a load on linguistic processing. In the collaborative condition, participants alternated between listening to words spoken by others and generating an appropriate word in response. In contrast, the individual condition required participants to repeatedly perform the generation task without attending to others. As the cognitive load associated with generating words is higher than that of simply listening (Boiteau et al. 2014; Sio et al. 2018), it is possible that individuals working alone experienced greater cognitive decline and a more pronounced FoLSE. However, due to the strict constraints of the word chain games, such as the requirements for semantic and phonological continuity and the avoidance of repetition, participants were compelled to actively monitor and process their partners’ utterances in order to generate appropriate responses. That is, non-speaking intervals did not function as rest periods. This is precisely a characteristic of collaborative contexts, in which participants must both comprehend others’ contributions and temporarily store information to retrieve suitable responses. Therefore, it is unlikely that participants used non-speaking periods to focus exclusively on the calculation task without engaging in linguistic processing. It is therefore important to replicate these findings in varying language processing contexts to verify the robustness of the observed effects.

Furthermore, the linguistic asymmetry evident in individual performance, in which interference was more pronounced in the Japanese condition than in the Chinese condition, was eliminated in collaborative contexts. This phenomenon can be explained from multiple perspectives. In the individual condition, participants likely encountered greater challenges in lemma retrieval when processing Japanese. As Levelt et al. (1999) posited, L2 processing inherently involves lemma retrieval, which imposes a substantial cognitive burden. Given that Japanese may have been a more demanding L2 for some participants than Chinese, the cognitive load in the Japanese condition was amplified, leading to greater interference. In contrast, collaboration fundamentally altered task dynamics and interaction patterns. The distribution of lexical responsibility transformed L2 processing from an individual burden into a collective effort. Groups exhibited lexical priming synergy, whereby each member’s lexical choices facilitated subsequent word retrieval, effectively lowering individual activation thresholds (Pickering and Garrod 2004).

Moreover, the social interaction and communication inherent in collaborative contexts likely contributed to the elimination of linguistic asymmetry (Knutsen et al. 2018). According to Knutsen et al. (2018), dialogue navigation in joint activities involves dynamic coordination, in which interlocutors continuously monitor and adjust their utterances based on mutual feedback to maintain shared understanding. In the present study, similar mechanisms were likely at play: through interaction, participants were able to clarify misunderstandings, provide hints, and propose alternative words. This feedback loop, absent in the individual condition, reduced cognitive load and helped equalize performance across the Japanese and Chinese conditions. In several cases, we observed that participants in the collaborative condition appeared to deliberately select easier words out of consideration for their partner’s emotional comfort or cognitive load. While this tendency was not consistent across all dyads, it suggests that affective and social factors may have contributed to reducing the foreign language burden (Huang 2023; Kalsoom et al. 2020).

This observation aligns with recent evidence that affective and social support can play a crucial role in mitigating the emotional strain associated with foreign language use. For instance, Huang (2023) demonstrated that peer support in online language learning reduces foreign language anxiety by enhancing learners’ self-efficacy, while Kalsoom et al. (2020) found that social support in English-as-a-foreign-language classrooms lowers foreign language anxiety and increases learners’ willingness to communicate. These findings suggest that affective and social factors may have contributed to reducing the foreign language burden in our collaborative condition. Consequently, the disparities in interference observed in individual performance were effectively diminished in the collaborative setting.

Additionally, there may have been differences in the levels of anxiety experienced by participants depending on whether they were solving problems individually or collaboratively. Yang (2017) examined Chinese international students in Japan and demonstrated that Japanese language anxiety (Motoda 2000), which consists of anxiety related to communication in Japanese, concerns about Japanese language proficiency, and tension in public situations, exacerbates the foreign language effect. This phenomenon can be attributed to the depletion of cognitive resources, as individuals allocate cognitive capacity to managing Japanese language anxiety, leaving fewer resources available for problem-solving (MacIntyre and Gardner 1994). In the present study, when participants encountered difficulties while using a foreign language, those in the collaborative group could rely on assistance from others, whereas those in the individual group had to resolve difficulties on their own. As a result, the individual group may have experienced higher levels of anxiety. This heightened anxiety could have influenced the magnitude of the foreign language effect.

## Conclusions, limitations and future directions

### Conclusions

While most prior research on the FoLSE has focused on individual task contexts (Lee et al. 2016; Takano and Noda 1993, 1995; Takano and Yagyu 2021; Wen et al. 2025; Yang and Inoue 2017), the present study expands the scope by investigating collaborative settings that involve intensive use of a foreign language. By simulating realistic group interactions, our findings provide greater ecological validity and offer novel insights into how collaboration can influence cognitive performance in foreign language conditions. Specifically, the results suggest that group-based tasks can alleviate the cognitive costs typically associated with foreign language processing, thereby attenuating the FoLSE.

These findings have important practical implications. They may inform the design of collaborative learning environments and multilingual workplace communication strategies aimed at reducing the FoLSE and enhancing cognitive efficiency. Identifying contextual conditions that buffer against foreign language–induced cognitive decline can offer valuable guidance for optimizing communication in both academic and professional multilingual settings.

### Limitations and future directions

Several limitations should be acknowledged. First, the experimental tasks were designed to isolate cognitive effects under controlled conditions and may not fully capture the complexity of real-world problem-solving. In naturalistic environments, language use is often fluid, supported by nonverbal cues and compensatory strategies (Gullberg 1998). It is important to note that, in the present study, collaboration was found to alleviate cognitive load; however, this effect was identified within a specific task context. In other task settings, collaboration may impose additional cognitive demands rather than reduce them. For example, if participants were asked to explain how a bicycle works, the individual condition (one person giving the full explanation) would likely be easier than a collaborative condition in which two people alternate word by word—especially in a foreign language. Future research should therefore examine the FoLSE in more ecologically valid contexts, such as workplace collaboration, academic discussions, or multilingual team decision-making, to assess the generalizability of the current findings.

Second, although the sample size was comparable to that of previous foundational studies (Takano and Noda 1995; Takano and Yagyu 2021), a larger sample would improve statistical power and enable a more robust evaluation of the observed effects. In addition, the generalizability of the present findings is limited by the fact that participants were predominantly unbalanced bilinguals. Given that prior studies have demonstrated that greater foreign language proficiency tends to attenuate the magnitude of the FoLSE (Lee et al. 2016; Yang, 2017; Yang and Inoue 2017), future research involving more balanced bilingual populations may produce different patterns of results.

Finally, while the findings indicate that collaboration reduces the FoLSE, the underlying mechanisms remain to be fully elucidated. One plausible explanation is that shared cognitive load in group contexts eases the mental burden of foreign language processing (Kirschner et al. 2011). Group dynamics may also enhance motivation, increase engagement, and foster a sense of shared responsibility, thereby reducing anxiety and supporting performance (Hänze and Berger 2007; Park and Hinsz 2015). Furthermore, verbal scaffolding within collaborative dialogue may allow participants to build on each other’s utterances and compensate for linguistic limitations more effectively (Chen and Lin 2021). To advance our understanding of these mechanisms, future research should explore how cognitive load, social interaction, and emotional factors jointly contribute to the FoLSE in collaborative contexts. The use of computer-based simulations may also allow for precise manipulation of interactional variables (Peterson 2010). For instance, implementing digital turn-taking mechanisms in individual conditions could help isolate the effects of language output frequency and task pacing, thereby enabling more controlled comparisons of individual versus collaborative performance.

## Data Availability

The datasets used and analyzed during the current study are available from the corresponding author on reasonable request.

## References

[CR1] Adams SJ, Roch SG, Ayman R (2005) Communication medium and member familiarity: The effects on decision time, accuracy, and satisfaction. Small Group Res 36(3):321–353. 10.1177/1046496405275232

[CR2] Almor A (2008) Why does language interfere with vision-based tasks? Exp Psychol 55(4):260–268. 10.1027/1618-3169.55.4.260 18683623 10.1027/1618-3169.55.4.260

[CR3] Baddeley A (2012) Working memory: Theories, models, and controversies. Ann Rev Psychol 63:1–29. 10.1146/annurev-psych-120710-100422 21961947 10.1146/annurev-psych-120710-100422

[CR4] Barr DJ, Levy R, Scheepers C, Tily HJ (2013) Random effects structure for confirmatory hypothesis testing: Keep it maximal. J Mem Lang 68(3):255–278. 10.1016/j.jml.2012.11.001 10.1016/j.jml.2012.11.001 PMC3881361 24403724

[CR5] Boiteau TW, Malone PS, Peters SA, Almor A (2014) Interference between conversation and a concurrent visuomotor task. J Exp Psychol Gen 143(1):295–311. 10.1037/a0031858 23421443 10.1037/a0031858 PMC3720820

[CR6] Bransford JD, Johnson MK (1972) Contextual prerequisites for understanding: Some investigations of comprehension and recall. J Verbal Learn Verbal Behav 11(6):717–726. 10.1016/S0022-5371(72)80006-9

[CR7] Cai F, Fei X, Song Q (2025) Japanese-as-a-foreign-language acquisition affects native Chinese lexical processing among Chinese learners. Front Psychol 15:1457155. 10.3389/fpsyg.2024.1457155 39850971 10.3389/fpsyg.2024.1457155 PMC11756525

[CR8] Chen Y-S, Lin M-F (2021) Effects of peer collaboration on EFL learners’ comprehension of conversational implicatures. System 97:102441. 10.1016/j.system.2020.102441

[CR9] R Core Team (2024) R: A Language and environment for statistical computing (version 4.4) (Computer software). Retrieved from https://cran.r-project.org. (R packages retrieved from CRAN snapshot. 2024/08/07)

[CR10] Costa A, Vives ML, Corey JD (2017) On language processing shaping decision making. Curr Dir Psychol Sci 26(2):146–151. 10.1177/0963721416680263

[CR11] Esmaeili Bijarsari SE (2021) A current view on dual-task paradigms and their limitations to capture cognitive load. Front Psychol 12:648586. 10.3389/fpsyg.2021.648586 34093335 10.3389/fpsyg.2021.648586 PMC8173029

[CR12] Fei X, Zhao S, Liu J (2022) Auditory recognition of Chinese-Japanese cognates and homographs by Chinese JFL learners. Psychologia 64(1):1–22. 10.2117/psysoc.2021-A144

[CR13] Gallucci M (2019) GAMLj: General analyses for linear models. [jamovi module]. Retrieved from https://gamlj.github.io/

[CR14] Gullberg M (1998) Gesture as a communication strategy in second language discourse: A study of learners of French and Swedish. In: McCafferty SG, Stam G (eds) Gesture: Second language acquisition and classroom research. Routledge, Abingdon, pp 109–130

[CR15] Hadjichristidis C, Geipel J, Surian L (2017) How foreign language affects decisions: Rethinking the brain-drain model. J Int Bus Stud 48(5):645–651. 10.1057/s41267-016-0040-1

[CR16] Hadjichristidis C, Geipel J, Keysar B (2019) The influence of native language in shaping judgment and choice. Prog Brain Res 247:253–272. 10.1016/bs.pbr.2019.02.003 31196437 10.1016/bs.pbr.2019.02.003

[CR17] Hänze M, Berger R (2007) Cooperative learning, motivational effects, and student characteristics: An experimental study comparing cooperative learning and direct instruction in 12th grade physics classes. Learn Instruction 17(1):29–41. 10.1016/j.learninstruc.2006.11.004

[CR18] Hayakawa S, Tannenbaum D, Costa A, Corey JD, Keysar B (2017) Thinking more or feeling less? Explaining the foreign-language effect on moral judgment. Psychol Sci 28(10):1387–1397. 10.1177/0956797617720944 28806137 10.1177/0956797617720944

[CR19] Huang Y (2023) Examining the relationship between peer support and foreign language emotions in online learning: The mediating effect of self-efficacy. Front Psychol 14:1148472. 10.3389/fpsyg.2023.1148472 37842693 10.3389/fpsyg.2023.1148472 PMC10572343

[CR20] Hutchins E (1995) Cognition in the wild. The MIT Press, Cambridge, MA. 10.7551/mitpress/1881.001.0001

[CR21] Ivaz L, Costa A, Duñabeitia JA (2016) The emotional impact of being myself: Emotions and foreign-language processing. J Exp Psychol Learn Mem Cognit 42(3):489–496. 10.1037/xlm0000179 26348199 10.1037/xlm0000179

[CR22] Jiao L, Wang X, Timmer K, Liu C (2025) The foreign language effect on moral judgement: Insights from the self–other moral bias. Int J Bilingual Educ Biling 28(4):495–506. 10.1080/13670050.2024.2445260

[CR23] Kalsoom A, Soomro NH, Pathan ZH (2020) How social support and foreign language anxiety impact willingness to communicate in English in an EFL classroom. Int J Engl Linguistics 10(2):80–91. 10.5539/ijel.v10n2p80

[CR24] Keysar B, Hayakawa SL, An SG (2012) The foreign-language effect: Thinking in a foreign tongue reduces decision biases. Psychol Sci 23(6):661–668. 10.1177/0956797611432178 22517192 10.1177/0956797611432178

[CR25] Kirschner PA (2002) Cognitive load theory: Implications of cognitive load theory on the design of learning (Editorial). Learn Instruction 12(1):1–10. 10.1016/S0959-4752(01)00014-7

[CR26] Kirschner F, Paas F, Kirschner PA (2011) Task complexity as a driver for collaborative learning efficiency: The collective working-memory effect. Appl Cogn Psychol 25(4):615–624. 10.1002/acp.1730

[CR27] Knutsen D, Col G, Le Bigot L (2018) An investigation of the determinants of dialogue navigation in joint activities. Appl Psycholinguist 39(6):1345–1371. 10.1017/S0142716418000358

[CR28] Kubose TT, Bock K, Dell GS, Garnsey SM, Kramer AF, Mayhugh J (2006) The effects of speech production and speech comprehension on simulated driving performance. Appl Cogn Psychol 20(1):43–63. 10.1002/acp.1164

[CR29] Kunar MA, Carter R, Cohen M, Horowitz TS (2008) Telephone conversation impairs sustained visual attention via a central bottleneck. Psychonomic Bull Rev 15(6):1135–1140. 10.3758/PBR.15.6.1135 10.3758/PBR.15.6.1135 PMC2684625 19001580

[CR30] Lee, C., Takano, Y., & Harada, Y. (2016). 英語力と外国語副作用との相関関係に関する検討 [Investigation on correlation between English proficiency and foreign language side effect] [Poster Session 3]. The 14th Conference of the Japanese Society for Cognitive Psychology, 132. doi:10.14875/cogpsy.2016.0_132

[CR31] Leone C, Feys P, Moumdjian L, D’Amico E, Zappia M, Patti F (2017) Cognitive-motor dual-task interference: a systematic review of neural correlates. Neurosci Biobehav Rev 75:348–360. 10.1016/j.neubiorev.2017.01.010 28104413 10.1016/j.neubiorev.2017.01.010

[CR32] Levelt WJM, Roelofs A, Meyer AS (1999) A theory of lexical access in speech production. Behav Brain Sci 22(1):1–38. 10.1017/s0140525x99001776. 11301520 10.1017/s0140525x99001776

[CR33] Li P, Zhang F, Yu A, Zhao X (2020) Language history questionnaire (LHQ3): An enhanced tool for assessing multilingual experience. Biling Lang Cogn 23(5):938–944. 10.1017/S1366728918001153

[CR34] Liu H, Cao F (2016) L1 and L2 processing in the bilingual brain: A meta-analysis of neuroimaging studies. Brain Lang 159:60–73. 10.1016/j.bandl.2016.05.013 27295606 10.1016/j.bandl.2016.05.013

[CR35] MacIntyre PD, Gardner RC (1994) The subtle effects of language anxiety on cognitive processing in the second language. Lang Learn 44(2):283–305. 10.1111/j.1467-1770.1994.tb01103.x

[CR36] Motoda S (2000) Measurement of second language anxiety in the target language environment: The Japanese language anxiety scale- Test construction, reliability, and validity. Japanese J Educational Psychol 48:422–432

[CR37] Nawal AF (2018) Cognitive load theory in the context of second language academic writing. High Educ Pedagogies 3(1):385–402. 10.1080/23752696.2018.1513812

[CR38] Norman DA, Bobrow DG (1975) On data-limited and resource-limited processes. Cogn Psychol 7(1):44–64. 10.1016/0010-0285(75)90004-3

[CR39] Paas F, Tuovinen JE, Tabbers H, Van Gerven PWM (2003) Cognitive load measurement as a means to advance cognitive load theory. Educational Psychol 38(1):63–71. 10.1207/S15326985EP3801_8

[CR40] Park ES, Hinsz VB (2015) Group interaction sustains positive moods and diminishes negative moods. Group Dynamics 19(4):290–298. 10.1037/gdn0000034 10.1037/gdn0000034 PMC4764092 26924925

[CR41] Peterson M (2010) Computerized games and simulations in computer-assisted language learning: A meta-analysis of research. Simul Gaming 41(1):72–93. 10.1177/1046878109355684

[CR42] Pickering MJ, Garrod S (2004) Toward a mechanistic psychology of dialogue. Behav Brain Sci 27(2):169–190. 10.1017/S0140525X04000056. 15595235 10.1017/s0140525x04000056

[CR43] Privitera AJ (2024) Influence of cognitive control on the moral foreign language effect. Int J Biling 29(5):1460–1473. 10.1177/13670069241292498

[CR44] Privitera AJ, Li S, Zhou Y, Wang M (2023) Modulatory role of foreign language experience on the moral foreign language effect. Biling Lang Cogn 26(5):1038–1050. 10.1017/S1366728923000275

[CR45] Purpuri S, Vasta N, Filippi R, Wei L, Mulatti C (2025) Does language shape the way we think? A review of the foreign language effect across domains. Int J Biling 29(1):269–291. 10.1177/13670069231225374

[CR46] Sio UN, Kotovsky K, Cagan J (2018) Silence is golden: The effect of verbalization on group performance. J Exp Psychol Gen 147(6):939–944. 10.1037/xge0000456 29888944 10.1037/xge0000456

[CR47] Song Q, Song T, Fei X (2023) Effects of executive functions on consecutive interpreting for Chinese-Japanese unbalanced bilinguals. Front Psychol 14:1236649. 10.3389/fpsyg.2023.1236649 37727743 10.3389/fpsyg.2023.1236649 PMC10506074

[CR48] Song Q, Fei X, Matsumi N (2025) Effects of input modality and second-language vocabulary proficiency on processing of Japanese compound verbs. Humanit Social Sci Commun 12(1):1–11. 10.1057/s41599-025-04649-7

[CR49] Storch N (2011) Collaborative writing in L2 contexts: Processes, outcomes, and future directions. Annu Rev Appl Linguist 31:275–288. 10.1017/S0267190511000079

[CR50] Swain M, Lapkin S (1998) Interaction and second language learning: Two adolescent French immersion students working together. Mod Lang J 82(3):320–337. 10.1111/j.1540-4781.1998.tb01209.x

[CR51] Tajima M, Sato N, Hashimoto M, Matsushita T, Sasao Y (2018) Development of the Japanese common academic word test. Bull Chuo-Gakuin University―Man Nat 45:19–31

[CR52] Takano Y, Noda A (1993) A temporary decline of thinking ability during foreign language processing. J Cross-Cult Psychol 24(4):445–462. 10.1177/0022022193244005

[CR53] Takano Y, Noda A (1995) Interlanguage dissimilarity enhances the decline of thinking ability during foreign language processing. Lang Learn 45(4):657–681. 10.1111/j.1467-1770.1995.tb00457.x

[CR54] Takano Y, Yagyu T (2021) Foreign language side effect when inner language is suspected to accompany thinking: Lowered thinking ability in daily verbal communication. Cogn Studies: Bull Japanese Cogn Sci Soc 28:271–281. 10.11225/cs.2021.001

[CR55] Takano Y, Yagyu T, Kishimoto K (2003) 外国語副作用—言語処理を伴う思考の場合 [Foreign language side effect: Case of thinking with language processing] [Poster Presentation 1: Memory, Learning, and Thinking]. Proceedings of the 1st Conference of the Japanese Society for Cognitive Psychology, 166. Doi:10.14875/cogpsy.2003.0.166.0

[CR56] Tyler MD (2001) Resource consumption as a function of topic knowledge in nonnative and native comprehension. Lang Learn 51(2):257–280. 10.1111/1467-9922.00155

[CR57] Wen X, Hirami M, Fujiki D (2025) Comparison of the effects of lexical retrieval and syntactic analysis on the occurrence of foreign language side effects. Japanese J Psychol 96(3):187–192. 10.4992/jjpsy.96.23330

[CR58] Yang, J. (2017). Gengonouryoku to gengohuan ga gaikokugohukusayou ni oyobosu eikyou [Effects of language ability and language anxiety on foreign language side effects]. Proceedings of the 59th conference of the Japanese Journal of Educational Psychology, 555. Doi:10.20587/pamjaep.59.0_555

[CR59] Yang J, Inoue W (2017) GaiKokugo fukusayowa zainichityugokujin ryugakusei niarawarerunoka [Foreign language side-effect in Chinese international students]. J Learn Sci 10:143–148. 10.15027/42648

[CR60] Zhang Y, Mi Y (2010) Another look at the language difficulties of international students. J Stud Int Educ 14(4):371–388. 10.1177/1028315309336031

